# Large out-of-plane spin–orbit torque in topological Weyl semimetal TaIrTe_4_

**DOI:** 10.1038/s41467-024-48872-3

**Published:** 2024-05-31

**Authors:** Lakhan Bainsla, Bing Zhao, Nilamani Behera, Anamul Md. Hoque, Lars Sjöström, Anna Martinelli, Mahmoud Abdel-Hafiez, Johan Åkerman, Saroj P. Dash

**Affiliations:** 1 Department of Microtechnology and Nanoscience, Chalmers University of Technology, SE-41296 Göteborg, Sweden; 2 Department of Physics, Indian Institute of Technology Ropar, Rupnagar, 140001 Punjab India; 3 Department of Physics, University of Gothenburg, Göteborg, SE-41296 Göteborg, Sweden; 4 Department of Chemistry and Chemical Engineering, Chalmers University of Technology, Göteborg, 41296 Sweden; 5 Department of Applied Physics and Astronomy, University of Sharjah, P. O. Box 27272, Sharjah, United Arab Emirates; 6 Department of Physics and Astronomy, Uppsala University, Box 516, SE-751 20 Uppsala, Sweden; 7 Center for Science and Innovation in Spintronics, Tohoku University, 2-1-1 Katahira, Aoba-ku, Sendai, 980-8577 Japan; 8 Research Institute of Electrical Communication, Tohoku University, 2-1-1 Katahira, Aoba-ku, Sendai, 980-8577 Japan; 9 Wallenberg Initiative Materials Science for Sustainability, Department of Microtechnology and Nanoscience, Chalmers University of Technology, SE-41296 Göteborg, Sweden; 10 Graphene Center, Chalmers University of Technology, SE-41296 Göteborg, Sweden

**Keywords:** Spintronics, Magnetic properties and materials

## Abstract

The unique electronic properties of topological quantum materials, such as protected surface states and exotic quasiparticles, can provide an out-of-plane spin-polarized current needed for external field-free magnetization switching of magnets with perpendicular magnetic anisotropy. Conventional spin–orbit torque (SOT) materials provide only an in-plane spin-polarized current, and recently explored materials with lower crystal symmetries provide very low out-of-plane spin-polarized current components, which are not suitable for energy-efficient SOT applications. Here, we demonstrate a large out-of-plane damping-like SOT at room temperature using the topological Weyl semimetal candidate TaIrTe_4_ with a lower crystal symmetry. We performed spin–torque ferromagnetic resonance (STFMR) and second harmonic Hall measurements on devices based on TaIrTe_4_/Ni_80_Fe_20_ heterostructures and observed a large out-of-plane damping-like SOT efficiency. The out-of-plane spin Hall conductivity is estimated to be (4.05 ± 0.23)×10^4^ (ℏ ⁄ 2*e*) (Ωm)^−1^, which is an order of magnitude higher than the reported values in other materials.

## Abstract (web summary)

Topological semimetals offer the potential for new-generation spintronic devices. Here, the authors demonstrate a large out-of-plane damping-like spin–orbit torque efficiency in a heterostructure based on the Weyl semimetal TaIrTe_4_.

## Introduction

Spin–orbit torque (SOT), utilizing the charge-to-spin conversion in a high spin–orbit coupling material (SOM) to create magnetization dynamics in an adjacent ferromagnet (FM), is expected to provide a breakthrough for next-generation memory and logic technologies^1,2^. SOT-based memory devices have the potential to challenge the devices based on spin-transfer torque, but the use of conventional SOMs leads to moderate efficiency. Furthermore, as the component of torque lies in-plane in conventional SOMs such as Pt and Ta, they are only suitable for deterministic switching of in-plane magnets^1–4^. However, for thermally stable high-density memory technologies, the industry requires magnets with perpendicular magnetic anisotropy (PMA), where additional measures, such as magnetic field assistance, will be required for deterministic switching. The first nontrivial requirement for a practical SOT memory technology is therefore the field-free deterministic SOT switching of FMs with PMA^2^.

To achieve this, SOMs with lower crystal symmetry are needed to generate out-of-plane damping-like torque components suitable for switching PMA ferromagnets. In contrast to conventional SOT, with a torque vector perpendicular to the plane of the electron’s motion and the electric field, unconventional SOT produces a tilted torque vector. Recent experiments demonstrated that van der Waals SOMs with reduced crystal symmetry, such as WTe_2_ in heterostructure with FMs, allow the generation of a nontrivial current-induced spin polarization with out-of-plane SOT symmetries^5–10^. More recently, field-free SOT switching has been reported using WTe_2_/Fe_3_GeTe_2_ heterostructures induced by out-of-plane SOT from WTe_2_^5,7,11,12^. However, the out-of-plane SOT strengths obtained using materials such as WTe_2_, MoTe_2_, NbSe_2_, Mn_2_Au, MnPd_3_, IrO_2_, etc. are an order of magnitude smaller than the conventional in-plane SOT component, making it challenging to realize energy-efficient SOT devices^5–9,11–21^. Therefore, it is crucial to discover new materials that exhibit large out-of-plane spin polarization and SOT components.

The topological Weyl semimetal candidate TaIrTe_4_ has gained significant attention as it shows the presence of bulk Weyl nodes and Fermi-arc surface states, which are unique band crossings in momentum space and useful spin textures that can give a variety of unusual electronic and charge-to-spin conversion properties^22–26^. The combination of topological spin textures and lower crystal symmetry hence makes TaIrTe_4_ a promising candidate for energy-efficient SOT devices. Here, using spin–torque ferromagnetic resonance (STFMR) and second harmonic measurements^3,27^ in TaIrTe_4_/Ni_80_Fe_20_ heterostructures^3,27–29^, we show a significant out-of-plane damping-like torque and a substantial out-of-plane spin Hall conductivity at room temperature.

## Results and discussion

**Fig. 1:**
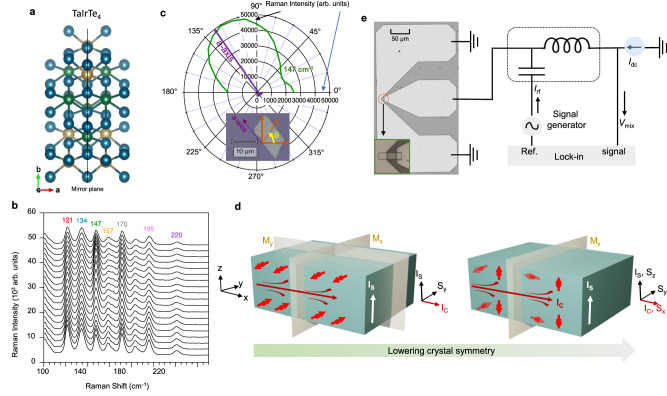
Crystal structure, the origin of unconventional spin–orbit torque, and schematic of the spin–torque ferromagnetic resonance (STFMR) measurement set-up with device geometry. **a** Crystal structure of *T*_d_-TaIrTe_4_ showing lower crystal symmetry with mirror plane along a-axis only. **b** Angle-dependent, polarized Raman spectra of TaIrTe_4_ obtained by rotating the polarization of the incident laser with respect to the sample that was fixed in position. **c** The representative polar plot of the angle-dependent intensity of A_g_ (A_1_) mode at ~147 cm^-1^ reveals a maximum value when laser polarization is aligned along the a-axis. **d** Conventional and unconventional SOT mechanisms in higher and lower crystalline symmetry materials. In high-symmetry materials, charge current *I*_C_, spin current *I*_S_ and spin polarization *S* (here *S*_y_) follow the conventional orthogonality relation (1^st^ diagram), while one can generate unconventional out-of-plane spin currents in lower symmetry materials where *I*_C_, *I*_S_ and spin polarization *S* do not follow the orthogonality relation as spin polarization also has a z-component (*S*_z_) (see 2^nd^ diagram; shown by removing *M*_y_ mirror plane). **e** Schematic of the spin–torque ferromagnetic resonance (STFMR) measurement set-up with TaIrTe_4_/Ni_80_Fe_20_ heterostructure device geometry. Scale bar is 50 μm for the device image, inset shows the magnified view of the microbar device.

TaIrTe_4_ is a promising topological Weyl semimetal candidate due to its large spin–orbit coupling and broken crystal symmetry, exhibiting unique chiral spin textures of electronic bands for bulk Weyl nodes and Fermi-arc surface states^30^. TaIrTe_4_ hosts only four type-II Weyl nodes providing the simplest model system with broken inversion symmetry. Additionally, the lower crystal symmetry of the *T*_d_-TaIrTe_4_ structure with space group *Pmn*2_1_ (Fig. 1a), can provide unconventional charge–spin conversion due to the presence of topologically non-trivial electronic states. The crystallographic alignment of the patterned devices is confirmed by performing angle-dependent, polarized Raman spectroscopy. Representative polarized Raman spectra are shown in Fig. 1b, the angle dependence is consistent with the one previously reported for *T*_d_-phase TaIrTe_4_^31^. The polar plot shown in Fig. 1c shows the integrated intensity of the A_g_(A_1_) Raman mode at a wavenumber of 147 cm^-1^. The intensity of this vibrational mode reaches a maximum when the laser polarization is parallel to the a-axis. These properties can result in the emergence of current-induced spin polarization that does not strictly follow the orthogonal relation between the charge current (*I*_C_), spin current (*I*_S_), and spin polarization (S) orientation as shown in Fig. 1d. The generation of an out-of-plane spin polarization can induce an out-of-plane SOT on the adjacent ferromagnet. In contrast to conventional in-plane SOT, which applies torque parallel to the device plane, the out-of-plane SOT can more efficiently manipulate magnetic moments with perpendicular components for high-density integration.

In this study, we focus on the unconventional charge-to-spin conversion in TaIrTe_4_, particularly the generation of out-of-plane damping-like SOT components. STFMR measurements are performed at room temperature to investigate the SOT in TaIrTe_4_/Ni_80_Fe_20_ bilayers. Ni_80_Fe_20_ is also known as permalloy (Py). The schematic of the STFMR measurement setup is shown in Fig. 1e. The STFMR microbars were fabricated using electron-beam lithography and argon ion milling. The devices were fabricated along the longer axis of the TaIrTe_4_ flakes, which is the a-axis in this class of materials^9,22,26^.

**Fig. 2:**
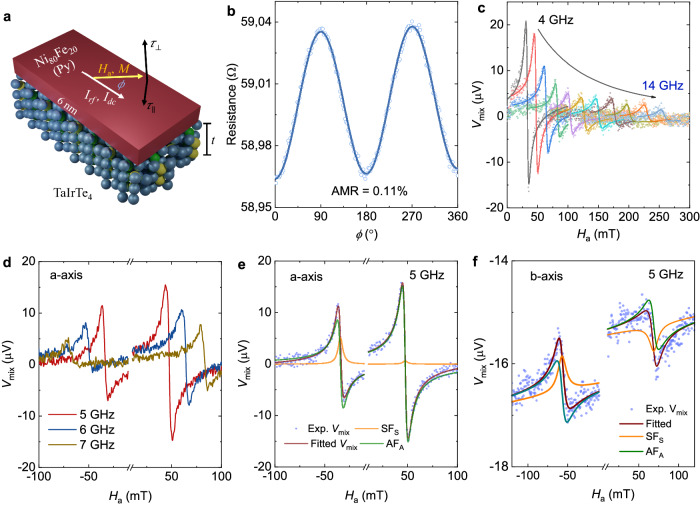
Unconventional charge–spin conversion in TaIrTe_4_. **a** Schematic of the TaIrTe_4_/Py heterostructure with SOT components, where *I*_rf_ and *I*_dc_ are the in-plane applied radio frequency and direct current, respectively. *H*_a_ applied magnetic field, *M* magnetization of the sample, and *ϕ* is the in-plane angle between applied current and magnetic field. ${\tau }_{\parallel }$ and ${\tau }_{{{{{{\rm{ \perp }}}}}}}$ are the in-plane and out-of-plane component of the damping-like torque. **b** Anisotropic magnetoresistance curve with an applied magnetic field of 100 mT and *dc* current of 0.6 mA. **c** Frequency-dependent STFMR spectra with in-plane magnetic field angle *ϕ* = 40° for a frequency range of 4-14 GHz. **d**, STFMR spectra with positive (*ϕ* = 40°) and negative (*ϕ* = 220°) applied magnetic field in the frequency range of 5–7 GHz. **e, f** The experimental STFMR curves (Exp. V_mix_), fitted curves using Eq. (1) (fitted V_mix_), symmetric (SF_S_) and antisymmetric (AF_A_) contributions in the V_mix_ (fitted) at 5 GHz for devices fabricated along a and b-axis respectively. In **c,** **e**, and **f** solid symbols represent the experimental STFMR data and solid lines are fit to the obtained data using Eq. (1). For **b–e** measurements are performed in the TaIrTe_4_(133 nm)/Py(6 nm) device fabricated along a-axis, while for **f** measurements are performed in TaIrTe_4_(30 nm)/Py(6 nm) device fabricated along b-axis.

**Fig. 3:**
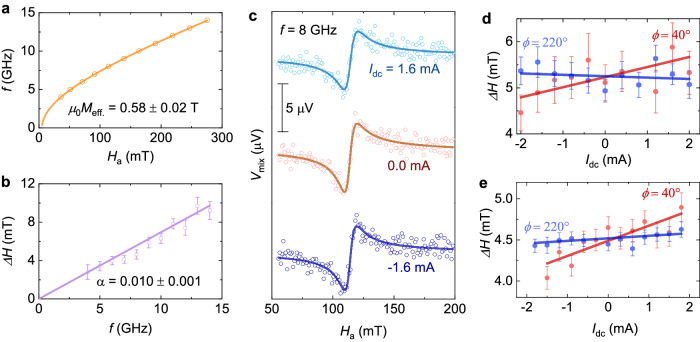
Evaluation of effective spin-orbit torque efficiency from *dc* bias dependence STFMR measurements. **a, b** Ferromagnetic resonance frequency *f vs*. resonance field *H*_R_ and ferromagnetic resonance linewidth *ΔH vs. f* for TaIrTe_4_(90 nm)/Py(6 nm) device, respectively. **c**, STFMR curves with different values of *dc* current (*I*_dc_) at 8 GHz, and **d,** *ΔH vs. I*_dc_ for TaIrTe_4_ (90 nm)/Py (6 nm) device, respectively. **e** *ΔH versus I*_dc_ for TaIrTe_4_(64 nm)/Py (5 nm) device at 7 GHz. In **a**–**b**, and **d**–**e**, solid symbols show the extracted values after fitting the experimental data to Eq. 1 and solid lines are fit to the obtained data. While error bars are obtained using the fitting of extracted data to Eq. 1. In **c**, solid symbols show the experimental data points and solid lines are fit to the data using Eq. 1.

In STFMR measurements, an in-plane radio frequency current *I*_rf_ is applied along the a-axis of TaIrTe_4_ while an in-plane magnetic field *H*_a_ is applied at an angle *ϕ* concerning the *I*_rf_, as shown in Fig. 2a. *I*_rf_ in TaIrTe_4_ generates a spin current in the z-direction, which is injected into the adjacent Py layer and excites the Py into a processional motion. Thanks to its anisotropic magnetoresistance (AMR), the resistance of Py oscillates with the same frequency as that of *I*_rf_, and produces a *dc* mixing voltage V_mix_, which is then measured using a lock-in amplifier. AMR is measured for a TaIrTe_4_(133 nm)/Py(6 nm) device, and a value of 0.11% is obtained as shown in Fig. 2b and in Supplementary Fig. 2 for other devices. Figure 2c shows the representative STFMR signals V_mix_ for the TaIrTe_4_(133 nm)/Py(6 nm) device at room temperature. The obtained *V*_mix_ signal is then fitted using the equation^3,32^,

1 $${V}_{{mix}}=S{F}_{S}\left({H}_{a}\right)+A{F}_{A}\left({H}_{a}\right)$$

where, ${F}_{S}\left({H}_{a}\right)=\frac{{\Delta H}^{2}}{\left[{\Delta H}^{2}+{\left({H}_{a}-{H}_{R}\right)}^{2}\right]}$ and ${F}_{A}\left({H}_{a}\right)={F}_{S}\left({H}_{a}\right)\left[\frac{\left({H}_{a}-{H}_{R}\right)}{\Delta H}\right]$ are symmetric and antisymmetric Lorentzian functions, respectively. *S* and *A* are the amplitudes of the symmetric *F*_S_ and the antisymmetric *F*_A_ signals and are proportional to the current-induced in-plane torque (${\tau }_{\parallel }$) and out-of-plane torque (${\tau }_{{{{{{\rm{ \perp }}}}}}}$), respectively. Here, *H*_a_, *ΔH*, and *H*_R_ refer to the applied external magnetic field, the ferromagnetic resonance linewidth, and the ferromagnetic resonance field, respectively. *H*_R_ and *ΔH* are extracted and the effective magnetization of the Py layer, *μ*_0_*M*_*eff*._, is determined by fitting *f vs*. *H*_R_ to the Kittel equation, $f=\left(\frac{\gamma }{2\pi }\right){\mu }_{0}\sqrt{\left({H}_{R}-{H}_{k}\right)\left({H}_{R}-{H}_{k}+{M}_{{eff}}\right)}$. The effective Gilbert damping constant *α* is obtained by a linear fit of *ΔH vs*. *f* using $\Delta H={\Delta H}_{0}+\frac{\left(2\pi \alpha f\right)}{\gamma }$^33^. The values for *μ*_0_*M*_*eff*_ and *α* for the TaIrTe_4_ (90 nm)/Py (6 nm) device are given in Figs. 3a and b, respectively. The *μ*_0_*M*_*eff*_ and *α* values for Py, using different thicknesses of TaIrTe_4_, are given in Supplementary Fig. 3. The obtained values of the *μ*_0_M_eff._ and *α* are comparable to literature values for Py~5 nm films^34,35^.

The strengths of the current-induced torques for different *ϕ* values are related to the symmetries of the device. For example, in conventional Pt/Py bilayers, the two-fold rotational symmetry requires that the SOT changes sign when the magnetization is rotated 180° in-plane, which results in the sign reversal of *V*_mix_ while retaining the same amplitude^5^. STFMR measurements with positive (*ϕ* = 40°) and negative (*ϕ* = 220°) applied fields were performed on the TaIrTe_4_/Py devices, and a clear change in both amplitude and shape are obtained as shown in Fig. 2d for the TaIrTe_4_(133 nm)/Py(6 nm) device, in the frequency range of 5-7 GHz. The *V*_mix_ signal is then fitted with Eq. (1) and the symmetric and antisymmetric components are obtained, which show a large change in amplitude in Fig. 2e for the TaIrTe_4_(133 nm)/Py(6 nm) device at 5 GHz. It directly indicates that the SOT is affected by the reduced symmetry of the TaIrTe_4_ layer and in particular the amplitude difference of the antisymmetric part indicates the clear presence of out-of-plane damping-like torque ${\tau }_{{{{{{\rm{ \perp }}}}}}}$. The change in amplitude for the symmetric part is also observed for the device measured in Fig. 2e, which indicates the presence of out-of-plane field-like torque in the system^36,37^ but it was absent in devices with other thicknesses. The STFMR measurements were also performed on the devices fabricated along the b-axis (current flows along the b-axis) of TaIrTe_4_, the representative curves are shown in Fig. 2f. The amplitude of *V*_mix_ is almost constant in the devices along the b-axis, which confirms that the reduced crystal symmetry along a-axis helps to generate out-of-plane damping-like SOT.

The charge-to-spin conversion efficiency for in-plane (***σ***_y_) and out-of-plane spin (***σ***_z_) is evaluated using the symmetric and antisymmetric amplitudes obtained with both positive and negative applied magnetic field *H*_a_ at a fixed value of *ϕ* (see Supplementary Note 1 for analysis details)^3,7,38^. The evaluated in-plane damping-like torque (${\xi }_{{DL},y}$) and out-of-plane damping-like torque (${\xi }_{{DL},z}$) efficiencies for TaIrTe_4_(133 nm)/Py(6 nm) and TaIrTe_4_(20 nm)/Py(6 nm) devices are plotted in Supplementary Fig. 4, which show enhancement in SOT efficiency as the *I*_rf_ frequency is increased. Such a frequency-dependent SOT efficiency has previously been observed in the WTe_2_ /NiFe system^7^. *ξ*_*DL,z*_ varies from 1 to 2.19, while ${\xi }_{{DL},y}$ varies from 0.36 to 0.63 for a frequency range of 5 to 10 GHz. However, as SOT efficiency evaluation using lineshape analysis can be affected by artifact voltages contributing towards *V*_mix_^32,39,40^, we use these analyses to primarily gain a qualitative sense of the in-plane and out-of-plane damping-like SOT components, and then use the more reliable method of *dc* bias linewidth modulation and angular STFMR data for SOT evaluation, as discussed below.

To more accurately characterize the SOT efficiency, *dc* bias-dependent STFMR measurements are done to estimate the effective damping-like torque efficiency^3,32,41^, and representative curves for different values of *dc* current (*I*_dc_) for TaIrTe_4_(90 nm)/Py(6 nm) are shown in Fig. 3c. The resonance linewidht, *ΔH*, is subsequently extracted for different *I*_dc_ values, and Fig. 3d, e show the resulting *ΔH vs. I*_dc_ plots for STFMR devices with TaIrTe_4_(90 nm)/Py(6 nm) and TaIrTe_4_(64 nm)/Py(5 nm), respectively. The slope [*δΔH/δ*(*I*_dc_)] of linearly fitted *ΔH vs I*_dc_ data indicates the strength of damping-like SOT, and we extract^3,29,32,41^,

2 $${\xi }_{{DL}}^{{eff}}=\frac{2e}{{{\hslash }}}\frac{\left({H}_{a}+0.5{M}_{{eff}}\right){\mu }_{0}{M}_{S}{t}_{{FM}}}{\sin \phi }\frac{\gamma }{2\pi f}\frac{\delta \Delta H}{\delta \left({I}_{{dc},{TaIrTe}4}\right)}{A}_{C}$$

with *ϕ* the azimuthal angle between *I*_dc_ and *μ*_0_*H*_a_, *M*_S_ = 6.4×10^5 ^A/m the saturation magnetization of the Py layer^3,42^, *t*_FM_ the thickness of the Py layer, $\frac{\gamma }{2\pi }$ the effective gyromagnetic ratio of Py, *e* the elementary charge, ℏ the reduced Planck’s constant, *I*_*dc,TaIrTe*4_ the current in the TaIrTe_4_ layer, and *A*_C_ the cross-sectional area of the STFMR microbars. The *I*_*dc,TaIrTe*4_ value is estimated by using the measured resistance of the device and the known resistance of the Py layer (*R*_Py_ = 329 Ω)^34,35^. The effective damping-like torque efficiencies of 0.98 ± 0.21 and 1.18 ± 0.54 are obtained for TaIrTe_4_ devices of 64, and 90 nm thicknesses, respectively. The effective spin Hall conductivity (${\sigma }_{{SHC}}={\sigma }_{c}{\xi }_{{DL}}^{{eff}}$) values are evaluated to be (7.31 ± 3.30)×10^4^ (ℏ/2e) (Ωm)^-1^, (1.91 ± 0.42)×10^4^ (ℏ/2e) (Ωm)^-1^, and (−12.86 ± 3.68) × 10^4^ (ℏ/2e) (Ωm)^-1^ for TaIrTe_4_ device of 90, 64, and 20 nm (see the data in Supplementary Fig. 5), respectively, using the electrical conductivity (*σ*_*c*_) values given in Supplementary Table 2 (obtained using the electrical conductivity of Py layer, ${\sigma }_{{Py}}=21.3\times {10}^{5}{\varOmega }^{-1}{m}^{-1}$). The obtained electrical conductivity values for TaIrTe_4_ are in close agreement with the earlier reports^23,26^.

**Fig. 4:**
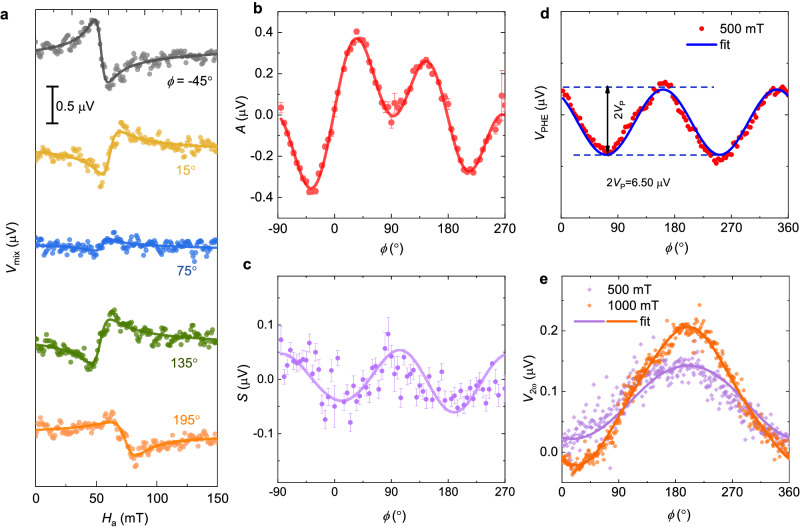
Unconventional spin–orbit torque in TaIrTe_4_ from angular STFMR and second harmonic Hall measurements. **a** The representative STFMR curves at different values of in-plane magnetic field angle *ϕ* for the frequency of 6 GHz. *ϕ* values are shown in the figure. **b, c** Antisymmetric (*A*) and symmetric (*S*) resonance amplitude versus *ϕ*. In both **b** and **c**, solid symbols represent the *A* and *S* values extracted after fitting the experimental data to Eq. (1), and solid lines are fit to the obtained data using Eqs. (3) and (4), respectively. Error bar in **b** and **c**, are obtained by fitting the experimental data to Eq. (1). The measurements are performed on the TaIrTe_4_(120 nm)/Py(6 nm) STFMR device. **d, e** Planar Hall voltage (*V*_*PHE*_) and second harmonic Hall signal (*V*_2*w*_) as a function of in-plane magnetic field angle, *ϕ*, for TaIrTe_4_(60 nm)/Py(6 nm) based Hall d**e**vices. In **d** and **e**, solid symbols are the experimental data points and solid lines are fit to the data.

As the amplitude of V_mix_ changes significantly with the sign of applied magnetic field in most of the devices (when *ϕ* is varied from 40° to 220°). This behavior of V_mix_ with *ϕ* indicates the presence of unconventional out-of-plane SOT in this system. To analyze this phenomenon in more detail, we performed angle-dependent STFMR measurements, where representative curves for sample TaIrTe_4_(120 nm)/Py(6 nm) are shown in Fig. 4a. The *S* and *A* values are extracted for different *ϕ* values and their angular dependence is plotted in Fig. 4b, c, respectively. In general, the spin current that generates torque has components along all three *x*, *y* and *z* axes, thus the allowed angular dependencies for the coefficients *S* and *A* are^3,5,28,43^,

3 $$S={S}_{{DL}}^{Y}{\cos\; \phi \; \sin} \, 2{\phi }+{S}_{{DL}}^{X}{\sin\; \phi \; \sin} \, 2{\phi }+{S}_{{FL}}^{Z}{\sin} \, 2{\phi }$$

4 $$A={A}_{{FL}}^{Y}{\cos\; \phi \; \sin} \, 2{\phi }+{A}_{{FL}}^{X}{\sin\; \phi \; \sin} \, 2{\phi }+{A}_{{DL}}^{Z}{\sin} \, 2\varphi$$

where ${S}_{{DL}}^{Y}$, ${S}_{{DL}}^{X}$, and ${A}_{{DL}}^{Z}$ are the coefficients for the damping-like torque with spin polarizations along *x*, *y*, and *z*, respectively; ${A}_{{FL}}^{Y}$, ${A}_{{FL}}^{X}$, and ${S}_{{FL}}^{Z}$ are the corresponding field-like torques. The angular dependence *S* and *A* is fitted using Eqs. (3) and (4) and the obtained parameters are given in Supplementary Table 1. While fits of the symmetric component *S* to Eq. 3 show moderate agreement, the extracted parameters are only required for the *y*- and *x*-polarized spin-current and do not contribute to the *z*-polarized spin-current estimation (see Eqs. 3–5 in Supplementary Note 2).

By considering that ${A}_{{FL}}^{Y}$ is due to the Oersted field alone, the amplitudes of the damping-like torque efficiencies per unit current density, ${\xi }_{{DL}}^{X}$, ${\xi }_{{DL}}^{Y}$, and ${\xi }_{{DL}}^{Z}$ in TaIrTe_4_ layer can be obtained (see Supplementary Note 2)^3,28^, ${\xi }_{{DL}}^{X}$, ${\xi }_{{DL}}^{Y}$, and ${\xi }_{{DL}}^{Z}$ values of 0.04 ± 0.01, 0.08 ± 0.02, and 0.11 ± 0.01 are obtained for the *t*_*TaIrTe*4_ = 120 nm sample. The spin Hall conductivity (${\sigma }_{{SHC}}^{k}={\sigma }_{c}{\xi }_{{DL}}^{k}$) can be evaluated using the electrical conductivity (*σ*_*c*_) values of TaIrTe_4_ (as given in Supplementary Table 1); ${\sigma }_{{SHC}}^{Z}=(4.05\pm 0.23)\times {10}^{4}$ (ℏ/2e) (Ωm)^-1^, ${\sigma }_{{SHC}}^{Y}=(2.87\pm 0.57)\times {10}^{4}$ (ℏ/2e) (Ωm)^-1^, and ${\sigma }_{{SHC}}^{X}=(1.43\pm 0.29)\times {10}^{4}$ (ℏ/2e) (Ωm)^-1^ are obtained for the 120 nm TaIrTe_4_ sample. The Dresselhaus-like symmetry torque (${\xi }_{{DL}}^{X}$) is present in this material system, this type of torque was earlier reported for both TaTe_2_/Py and WTe_2_/Py heterostructures^17^, and it appears due to the Orested field generated by a component of current flowing transverse to the applied voltage. The in-plane resistivity anisotropy of materials like WTe_2_ generates such spatially nonuniform current flow in the heterostructures. To investigate whether non-uniformity in the TaIrTe_4_ thickness makes any difference to the SOT efficiency, STFMR devices were made on such flakes (see Supplementary Fig. 6). Interestingly, ${\xi }_{{DL}}^{Z}$ shows sign reversal and a value of -0.07 is obtained for this device where the average thickness of TaIrTe_4_ is 64 nm.

To further confirm the out-of-plane damping-like SOT efficiency, the harmonic Hall measurements are performed as the STFMR method sometimes gives overestimated values^21^. Low-frequency alternating current (*I*_ac_) is applied to the devices, and second harmonic Hall voltages as a function of the angle of in-plane magnetic field angle, *ϕ*, is measured as shown in Fig. 4e. The damping-like SOT efficiency is estimated using fits of the second harmonic Hall voltage (*V*_2*w*_) *versus ϕ*, to^5,28,44,45^

5 $${V}_{2w}=	{D}_{{DL}}^{Y}\cos {\phi }+{D}_{{DL}}^{X}\sin {\phi }+{D}_{{DL}}^{Z}\cos 2{\phi }+{F}_{{FL}}^{Y}\cos {\phi \; cos}2{\phi } \\ 	+ {F}_{{FL}}^{X}\sin {\phi \; cos}2{\phi }+{F}_{{FL}}^{Z}$$

where ${D}_{{DL}}^{X}$, ${D}_{{DL}}^{Y}$, and ${D}_{{DL}}^{Z}$ are the coefficients of damping-like torque with spin polarizations along the *x*, y, and *z*, respectively. While, ${F}_{{FL}}^{X}$, ${F}_{{FL}}^{Y}$, and ${F}_{{FL}}^{Z}$ are the field-like torque counterparts. The damping-like torque efficiency is estimated as given in Supplementary Note 3, and an out-of-plane damping-like torque efficiency (${\theta }_{{DL}}^{Z}$) of 0.11 ± 0.06 is obtained which is in good agreement with the ${\xi }_{{DL}}^{Z}$ values obtained using the STFMR method.

In summary, we have demonstrated a large unconventional out-of-plane SOT efficiency in the Weyl semimetal candidate TaIrTe_4_ at room temperature. Using the STFMR and second harmonic Hall measurements of TaIrTe_4_/Py heterostructure devices, we observed a large damping-like torque due to efficient charge-to-spin conversion. From angular dependent STFMR measurements, the evaluated out-of-plane damping-like torque and the spin Hall conductivity (SHC) ${\sigma }_{{SHC}}^{Z}$ and ${\sigma }_{{SHC}}$ values are found to be an *order of magnitude* higher than many transition metal dichalcogenides (TMDs)^13,18,46^ including WTe_2_^5,7^, and comparable to the conventional SOT materials such as Bi_2_Se_3_^47^, and heavy metal Pt^3^. In contrast to conventional materials, TaIrTe_4_ also exhibits a very large out-of-plane SHC ${\sigma }_{{SHC}}^{Z}$. Utilization of such topological quantum materials with lower crystal symmetries and useful spin textures are therefore suitable for obtaining large charge-to-spin conversion efficiencies with unconventional out-of-plane SOT components. The antisymmetric SOT component in TaIrTe_4_ has the potential to realize spintronic technologies by enabling efficient manipulation of magnetic materials with perpendicular magnetic anisotropy, leading to high-density, faster, and energy-efficient memory, logic and communication technologies.

*Note*: During the review process of our manuscript, two other papers were also published reporting an out-of-plane SOT in TaIrTe_4_^48,49^.

## Methods

### Single crystal growth

TaIrTe_4_ crystals were grown by evaporating tellurium from a tellurium-iridium-tantalum melt. The temperature of the melt and crystal growth was 850 °C. The temperature at which tellurium condensed was 720 °C^50^. The chemical composition was studied with a digital scanning electronic microscope TESCAN Vega II XMU with energy dispersive microanalysis system INCA Energy 450/XT (20 kV). The EDX analysis showed that the approximate chemical composition of the samples was close to stoichiometric. No impurity elements and phases were found, see Supplementary Fig. 1 for details.

### Device fabrication

The samples were prepared by mechanically exfoliating nanolayers of TaIrTe_4_ crystals onto a SiO_2_/Si wafer by the Scotch tape method inside a glove box. To make TaIrTe_4_/Ni_80_Fe_20_ heterostructures, the samples were quickly transferred from the glove box to the high vacuum sputtering chamber to deposit Py(5-6 nm)/Al_2_O_3_(4 nm) layers. Ni_80_Fe_20_ is known as permalloy (Py). The sample surface was bias sputter cleaned for 20 sec with low energy Ar ion, followed by the deposition of 6 nm Py and 4 nm Al_2_O_3_ layers using the dc and rf magnetron sputtering methods, respectively. The TaIrTe_4_/Py heterostructures were patterned into the rectangular microstrips and Hall devices for spin–torque ferromagnetic resonance (STFMR) and harmonic Hall measurements, respectively, using the electron-beam lithography and Ar ion milling using the negative e-beam resist as the etching mask. Laser writer lithography was used to prepare the top co-planar waveguide contacts for electrical measurements, followed by the lift-off process of 200 nm of copper (Cu) and 20 nm of platinum (Pt).

### Polarized Raman spectroscopy

Polarized, angle-dependent Raman spectra were recorded using an InVia Raman spectrometer from Renishaw. A laser with a wavelength of 785 nm was used as the incident light, using a x50 Leica objective, a 1200 l/mm grating, in the back-scattering mode. The polarization of the laser was rotated from 0° to 180 ° at angle steps of 10 degrees, while keeping the sample specimen fixed with respect to the coordinate axes of the laboratory; no analyser was used along the path of the back-scattered light. The recorded Raman spectra were first analyzed as conventional linear plots (intensity vs. Raman shift, as in Fig. 1b) and successfully as polar plots in which the plotted intensities are integrated areas under selected peaks (intensity vs polarization angle, as in Fig. 1c).

### Anisotropic magnetoresistance measurements

In-plane angular dependence anisotropic magnetoresistance (AMR) measurements were performed on ST-FMR microbars using a rotatable projected vector field magnet with a fixed applied magnetic field of 0.1 T and applied dc current of 0.5 mA. The resistance of the devices was measured while rotating the magnetic field in-plane.

### Spin–orbit torque ferromagnetic resonance (STFMR) measurements

STFMR measurements were performed at room temperature on the microbar devices to estimate the spin–orbit torque (SOT) efficiency and other magneto dynamical parameters. The measurements for effective SOT analysis were performed with a fixed in-plane angle *ϕ* = 40°, the radio-frequency (rf) current modulated at 98.76 Hz was applied to the device through a high-frequency bias-T at a fixed frequency (ranging over 3-14 GHz) with an input rf power *P* = 14 dBm. The in-plane angle *ϕ* dependence STFMR measurements were performed using a rotatable projected field magnet with *ϕ* = 0-360° (step of 5°) at a fixed frequency.

### Harmonic Hall measurements

The schematic of the harmonic measurement setup is given elsewhere^29^, where a 213 Hz alternating current (*I*_ac_) is applied to the channel in the presence of a fixed applied magnetic field, ${\mu }_{0}{H}_{a}$. The second harmonic signal is measured while sweeping the in-plane angle, *ϕ*, between ${\mu }_{0}{H}_{a}$ and *I*_ac_. Further details of the measurements and analysis are provided in Supplementary Note 3.

### Supplementary information

Supplementary Information

Peer Review File

## Data Availability

The data that support the findings of this study are available from the corresponding authors on request.

## References

[1] Shao Q (2021). Roadmap of Spin–Orbit Torques. IEEE Trans. Magn..

[2] Manchon A (2019). Current-induced spin–orbit torques in ferromagnetic and antiferromagnetic systems. Rev. Mod. Phys..

[3] Liu L, Moriyama T, Ralph DC, Buhrman RA (2011). Spin–torque ferromagnetic resonance induced by the spin Hall effect. Phys. Rev. Lett..

[4] Liu L (2012). Spin–torque switching with the giant spin Hall effect of tantalum. Science.

[5] MacNeill D (2016). Control of spin–orbit torques through crystal symmetry in WTe2/ferromagnet bilayers. Nat. Phys..

[6] MacNeill, D. et al. Thickness dependence of spin–orbit torques generated by WTe2. *Phys. Rev. B Condens. Matter.* **96**, 054450 (2017).

[7] Shi S (2019). All-electric magnetization switching and Dzyaloshinskii–Moriya interaction in WTe2/ferromagnet heterostructures. Nat. Nanotechnol..

[8] Zhao B (2020). Unconventional charge–spin conversion in Weyl-semimetal WTe2. Adv. Mater..

[9] Yang H (2022). Two-dimensional materials prospects for non-volatile spintronic memories. Nature.

[10] Wieder BJ (2021). Topological materials discovery from crystal symmetry. Nat. Rev. Mater..

[11] Kao I-H (2022). Deterministic switching of a perpendicularly polarized magnet using unconventional spin–orbit torques in WTe2. Nat. Mater..

[12] Shin I (2022). Spin–orbit Torque Switching in an All-Van der Waals Heterostructure. Adv. Mater..

[13] Liang S (2020). Spin–orbit torque magnetization switching in MoTe2 /Permalloy heterostructures. Adv. Mater..

[14] Vila M (2021). Low-symmetry topological materials for large charge-to-spin interconversion: The case of transition metal dichalcogenide monolayers. Phys. Rev. Res.

[15] Liu L (2021). Symmetry-dependent field-free switching of perpendicular magnetization. Nat. Nanotechnol..

[16] Kurebayashi H, Garcia JH, Khan S, Sinova J, Roche S (2022). Magnetism, symmetry and spin transport in van der Waals layered systems. Nat. Rev. Phys..

[17] Stiehl GM (2019). Current-Induced Torques with Dresselhaus Symmetry Due to Resistance Anisotropy in 2D Materials. ACS Nano.

[18] Guimarães MHD, Stiehl GM, MacNeill D, Reynolds ND, Ralph DC (2018). Spin–orbit Torques in NbSe2/Permalloy Bilayers. Nano Lett..

[19] Dc M (2023). Observation of anti-damping spin–orbit torques generated by in-plane and out-of-plane spin polarizations in MnPd3. Nat. Mater..

[20] Patton M (2023). Symmetry control of unconventional spin–orbit torques in IrO2. Adv. Mater..

[21] Chen X (2021). Observation of the antiferromagnetic spin Hall effect. Nat. Mater..

[22] Ma J (2019). Nonlinear photoresponse of type-II Weyl semimetals. Nat. Mater..

[23] Jian Y (2020). Transport signatures of temperature-induced chemical potential shift and Lifshitz transition in layered type-II Weyl semimetal TaIrTe4. 2D Mater..

[24] Xing Y (2020). Surface superconductivity in the type II Weyl semimetal TaIrTe4. Natl Sci. Rev..

[25] Zhuo X (2021). Dynamical evolution of anisotropic response of type-II Weyl semimetal TaIrTe4 under ultrafast photoexcitation. Light Sci. Appl.

[26] Kumar D (2021). Room-temperature nonlinear Hall effect and wireless radiofrequency rectification in Weyl semimetal TaIrTe4. Nat. Nanotechnol..

[27] Tulapurkar AA (2005). Spin–torque diode effect in magnetic tunnel junctions. Nature.

[28] Bose A (2022). Tilted spin current generated by the collinear antiferromagnet ruthenium dioxide. Nat. Electron..

[29] Bainsla L. et al. Berry curvature induced giant intrinsic spin–orbit torque in single layer magnetic Weyl semimetal thin films. arXiv:2311.08145 (2023).

[30] Koepernik K., Kasinathan D., Efremov D. V., & Khim S. A ternary type-II Weyl semimetal. *Phys Rev B: Condens Matter Mater Phys*. **93**, 201101(R) (2016).

[31] Liu Y (2018). Raman signatures of broken inversion symmetry and in-plane anisotropy in type-II Weyl semimetal candidate TaIrTe4. Adv. Mater..

[32] Demasius K-U (2016). Enhanced spin–orbit torques by oxygen incorporation in tungsten films. Nat. Commun..

[33] Bainsla L (2022). Ultrathin ferrimagnetic GdFeCo films with low damping. Adv. Funct. Mater..

[34] Haidar M (2021). Compositional effect on auto-oscillation behavior of Ni100-xFex/Pt spin Hall nano-oscillators. Appl Phys. Lett..

[35] Haidar M (2019). A single layer spin–orbit torque nano-oscillator. Nat. Commun..

[36] Gupta P (2024). Generation of out-of-plane polarized spin current in (permalloy, Cu)/EuS interfaces. Phys. Rev. B Condens Matter.

[37] Hazra BK (2023). Generation of out-of-plane polarized spin current by spin swapping. Nat. Commun..

[38] Shi S. et al. Observation of the out‐of‐plane polarized spin current from CVD grown WTe 2. *Adv. Quant. Technol.* **4**, 2100038 (2021)

[39] Tserkovnyak Y, Brataas A, Bauer GEW (2002). Enhanced gilbert damping in thin ferromagnetic films. Phys. Rev. Lett..

[40] Saitoh E, Ueda M, Miyajima H, Tatara G (2006). Conversion of spin current into charge current at room temperature: Inverse spin-Hall effect. Appl Phys. Lett..

[41] Behera N. et al. Energy-efficient W100−xTax/ co-Fe-B/MgO spin hall nano-oscillators. *Phys. Rev. Appl.* **18**, 024017 (2022).

[42] Krivorotov IN (2005). Time-domain measurements of nanomagnet dynamics driven by spin-transfer torques. Science.

[43] Fang D (2011). Spin–orbit-driven ferromagnetic resonance. Nat. Nanotechnol..

[44] Hayashi M, Kim J, Yamanouchi M, Ohno H (2014). Quantitative characterization of the spin–orbit torque using harmonic Hall voltage measurements. Phys. Rev. B Condens Matter.

[45] Avci CO (2014). Interplay of spin–orbit torque and thermoelectric effects in ferromagnet/normal-metal bilayers. Phys. Rev. B Condens Matter.

[46] Safeer CK (2019). Room-temperature spin Hall effect in graphene/MoS2 van der Waals heterostructures. Nano Lett..

[47] Han J (2017). Room-Temperature Spin–orbit Torque Switching Induced by a Topological Insulator. Phys. Rev. Lett..

[48] Liu Y. et al. Field-free switching of perpendicular magnetization at room temperature using out-of-plane spins from TaIrTe4. *Nat. Electron.* **6**, 732–738 (2023).

[49] Zhang Y (2023). Room temperature field-free switching of perpendicular magnetization through spin–orbit torque originating from low-symmetry type II Weyl semimetal. Sci. Adv..

[50] Chareev DA (2020). Growth of transition-metal dichalcogenides by solvent evaporation technique. Cryst. Growth Des..

