# Supplementary material for: Large out-of-plane spin–orbit torque in topological Weyl semimetal TaIrTe4
Source: Nat Commun. 2024 May 31;15:4649. doi: 10.1038/s41467-024-48872-3 (PMC11143358; doi:10.1038/s41467-024-48872-3)
Supplement: Supplementary file 1 — Supplementary Information [file 41467_2024_48872_MOESM1_ESM.pdf]

Supplementary Information

Large out-of-plane spin-orbit torque in topological Weyl semimetal $TaIrTe_4$

Lakhan Bainsla,[1,2,*] Bing Zhao,[1] Nilamani Behera,[3] Anamul Md. Hoque,[1] Lars Sjöström,[1] Anna Martinelli,[4] Mahmoud Abdel-Hafiez,[5,6] Johan Åkerman,[3,7,8] Saroj P. Dash[1,9,10,*]

[1]*Department of Microtechnology and Nanoscience, Chalmers University of Technology,SE-41296, Göteborg, Sweden*
[2]*Department of Physics, Indian Institute of Technology Ropar, Rupnagar 140001, Punjab, India*
[3]*Department of Physics, University of Gothenburg, Göteborg, SE-41296, Göteborg, Sweden*
[4]*Department of Chemistry and Chemical Engineering, Chalmers University of Technology, Göteborg, 41296 Sweden*
[5]*Department of Applied Physics and Astronomy, University of Sharjah, P. O. Box 27272 Sharjah, United Arab Emirates*
[6]*Department of Physics and Astronomy, Uppsala University, Box 516, SE-751 20 Uppsala, Sweden*
[7]*Center for Science and Innovation in Spintronics, Tohoku University, 2-1-1 Katahira, Aoba-ku, Sendai 980-8577 Japan*
[8]*Research Institute of Electrical Communication, Tohoku University, 2-1-1 Katahira, Aoba-ku, Sendai 980-8577 Japan*
[9]*Wallenberg Initiative Materials Science for Sustainability, Department of Microtechnology and Nanoscience, Chalmers University of Technology,SE-41296, Göteborg, Sweden.*
[10]*Graphene Center, Chalmers University of Technology, SE-41296, Göteborg, Sweden*

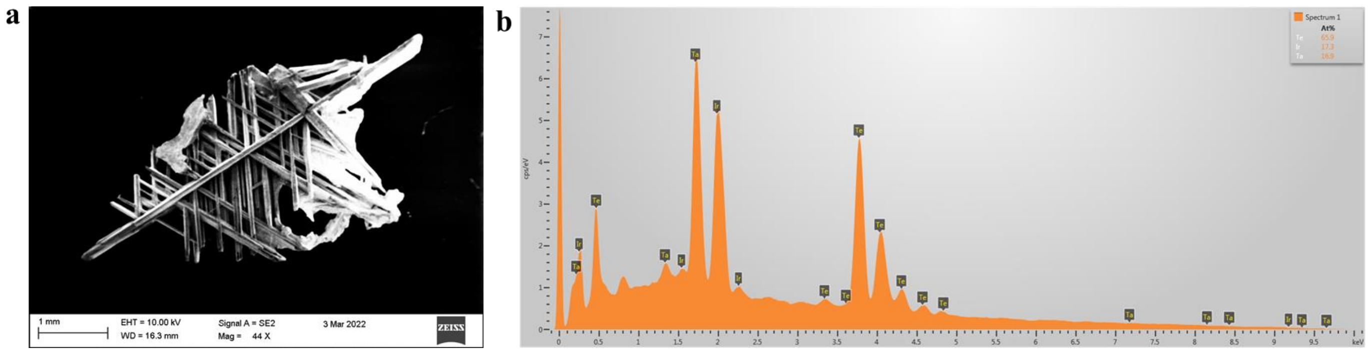

Supplementary Figure 1. Analysis of $TaIrTe_4$ single crystal. **a,** Scanning electron microscope image of $TaIrTe_4$ crystal, and **b,** energy dispersive X-ray (EDX) analysis of the crystals.

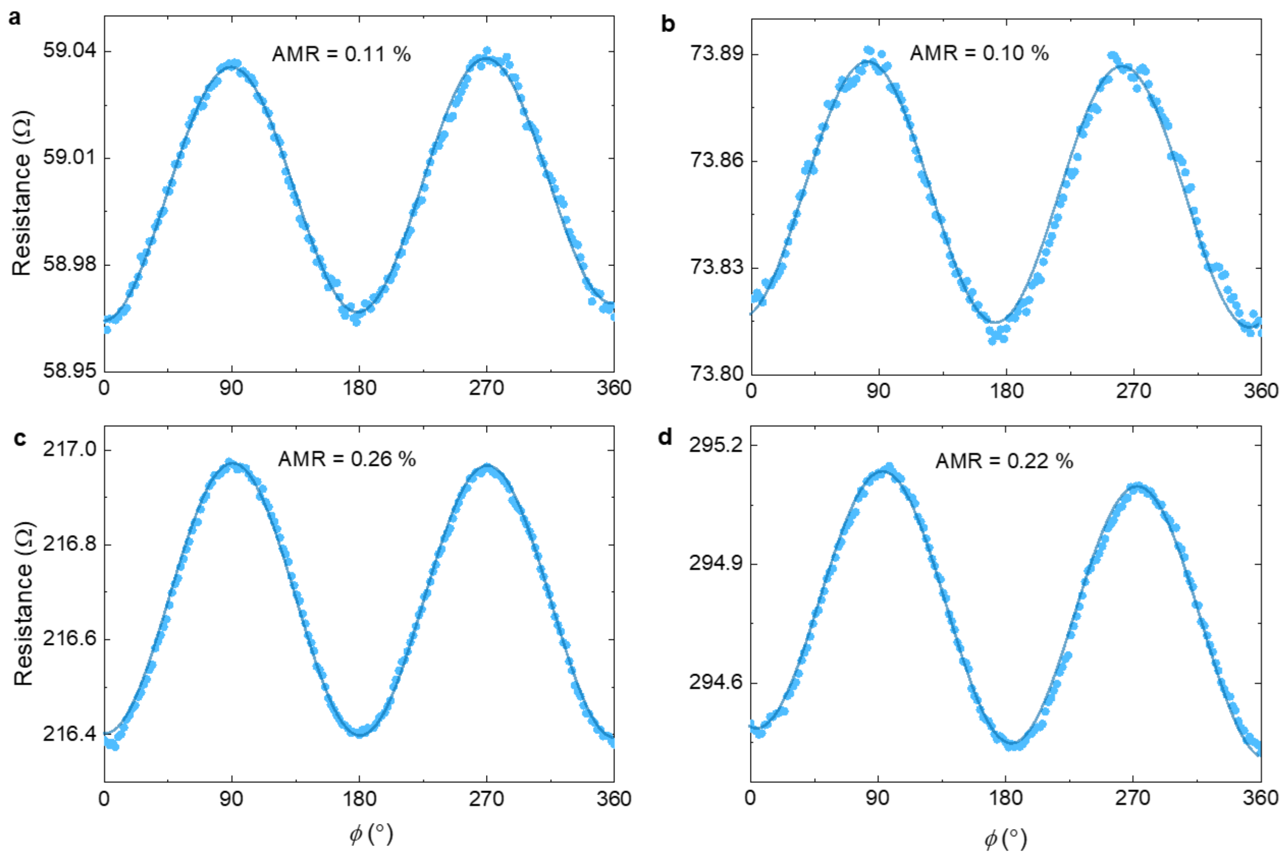

Supplementary Figure 2. **Anisotropic magnetoresistance (AMR) measurements on STFMR devices**. **a-c,** The AMR curves for $TaIrTe_4$(133 nm)/Py(6 nm), $TaIrTe_4$(120 nm)/Py(6 nm), and $TaIrTe_4$(90 nm)/Py(6 nm), respectively. **d,** AMR curve for $TaIrTe_4$(64 nm)/Py(5 nm) based device. AMR values for each device are given in the inset of the figures. Here, solid symbols represent the experimental data and solid lines are cosine fits to the data.

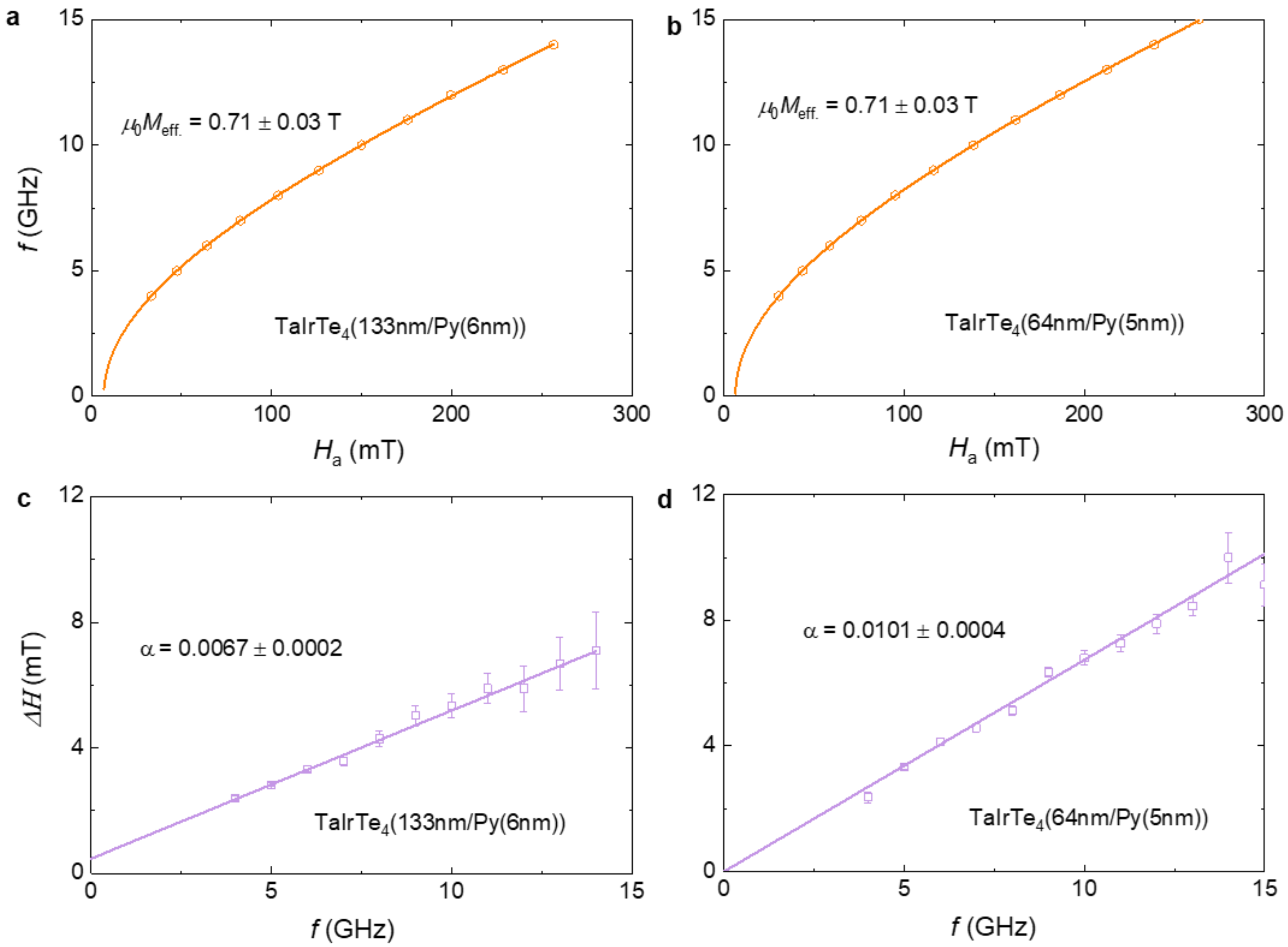

Supplementary Figure 3. **Effective magnetization** $\mu_0 M_{eff}$ **and Gilbert damping constant** α **analysis for** $\mathbf{TaIrTe_4}$**/Py samples**. **a, c,** Frequency f versus resonance field H_R, **b, d,** linewidth ΔH versus frequency data extracted from STFMR analysis for $TaIrTe_4$(133 nm)/Py(6 nm) and $TaIrTe_4$(64 nm)/Py(5 nm) devices, respectively. The value of effective magnetization and Gilbert damping constant is given in the inset of the figures. Here, open symbols represent the data values extracted by fitting the STFMR curves to Eq. (1) in manuscript and sold lines are fits to the data as mentioned in the manuscript. Error bars are obtained by fitting the experimental data to Eq. (1).

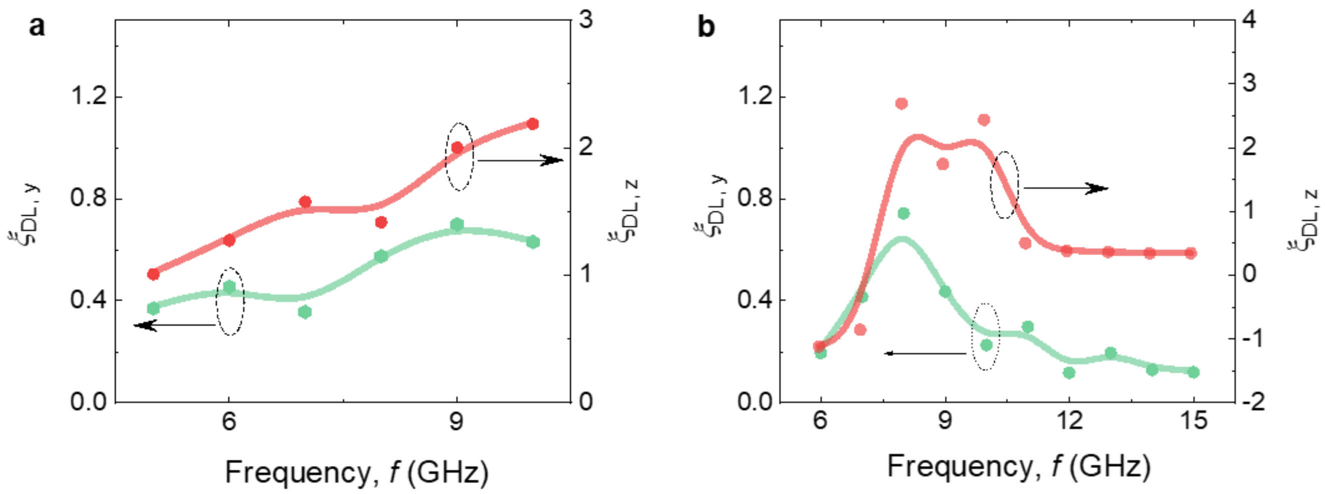

Supplementary Figure 4. a and **b,** Evaluated in-plane and out-of-plane damping-like SOT efficiencies, $\xi_{DL,y}$ and $\xi_{DL,z}$ using lineshape analysis at different frequencies for $TaIrTe_4$(133 nm)/Py(6 nm) and $TaIrTe_4$(20 nm)/Py(6 nm) STFMR devices, respectively. Here, solid symbols are the estimated efficiency values and solid lines are guides to the eye.

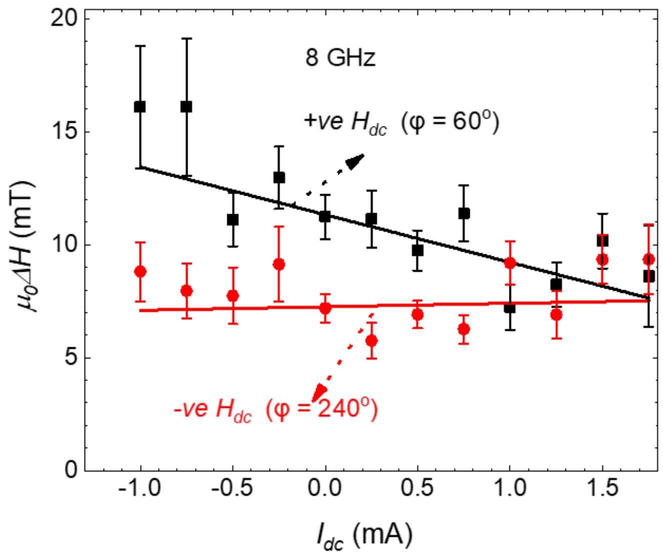

Supplementary Figure 5. *ΔH versus* I_{dc} for $TaIrTe_4$(20 nm)/Py (6 nm) device at 8 GHz. Here, solid symbols show the extracted values after fitting the experimental data to Eq. 1 and solid lines are fit to the obtained data. Error bars are obtained by fitting the experimental data to Eq. (1) in manuscript.

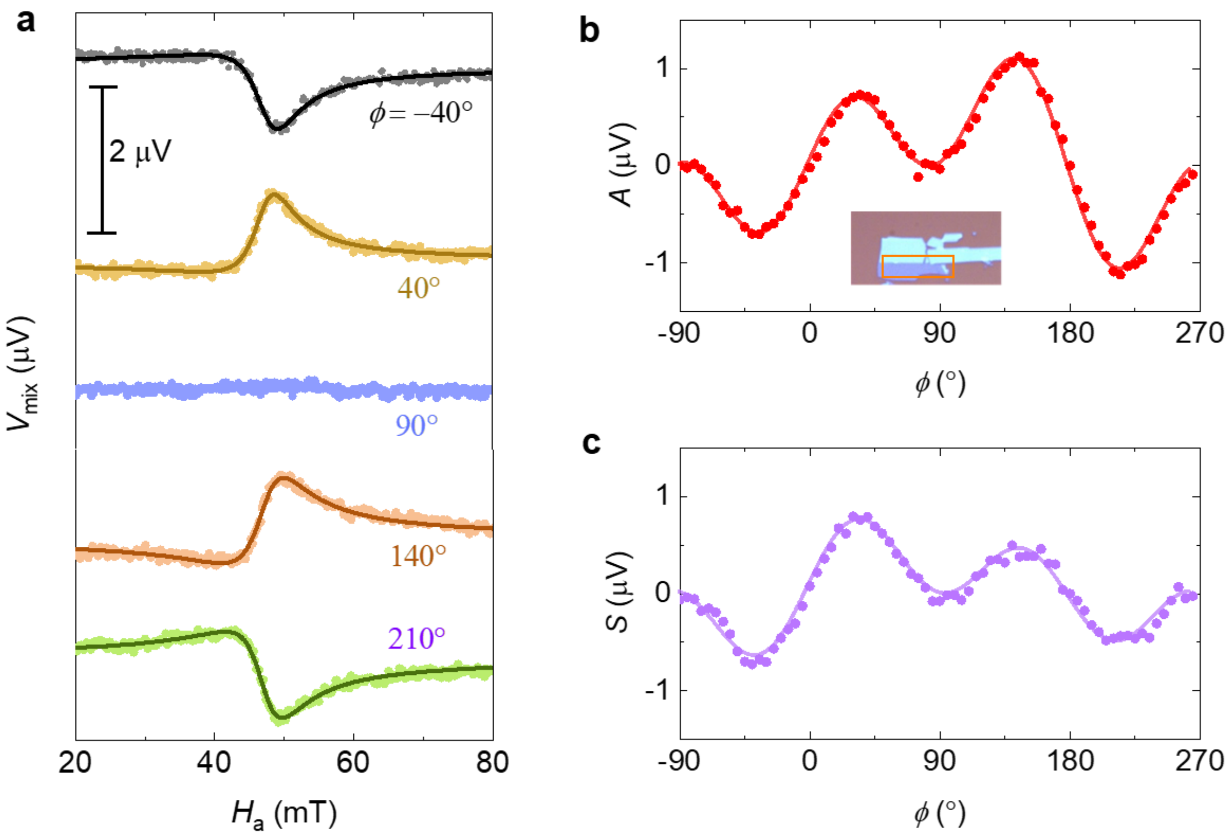

Supplementary Figure 6. Unconventional SOT in non-uniform $TaIrTe_4$ layer from angular STFMR measurements. a, The representative STFMR curves at different values of in-plane magnetic field angle, ϕ, at the frequency 6 GHz. ϕ values are shown in the figure. **b,** Antisymmetric resonance amplitude (A) versus ϕ and **c,** Symmetric component of resonance amplitude (S) versus ϕ. In both b and c solid symbols represent the A and S values extracted after fitting the experimental data to Eq. (1) in manuscript, and solid lines are fit to the obtained data using Eqs. (3) and (4), respectively. The measurements are performed in $TaIrTe_4$(64 nm)/Py(5 nm) device. Error bar in **b** and **c**, are obtained by fitting the experimental data to equation (1).

Supplementary Table 1. Damping-like torque efficiencies estimated using the extracted parameters from angular STFMR data.

Samples	A_{DL}^{Z}	S_{DL}^{Y}	S_{DL}^{X}	A_{FL}^{Y}	$\mu_0 M_S$ (T)	M_{eff} (T)	H_a (T)	ξ_{DL}^{Z}	ξ_{DL}^{Y}	ξ_{DL}^{x}
TaIrTe4(64nm) /Py(5nm)	-0.2±0.007	0.76±0.014	0.06±0.02	1.15±0.019	0.81	0.71	0.060	-0.07±0.01	0.97±0.02	0.07±0.02
TaIrTe4(120nm) /Py(6nm)	0.05±0.003	0.01	0.01	0.40±0.01	0.81	0.72	0.059	0.11±0.01	0.08±0.02	0.04±0.01

Supplementary Table 2. Thickness dependence of $TaIrTe_4$ electrical conductivity value extracted using the device resistance and the known resistance value of $Ni_{80}Fe_{20}$.

$TaIrTe_4$ thickness (nm)	$TaIrTe_4$ electrical conductivity ($10^5\ \Omega^{-1}m^{-1}$)
20	0.70
64	0.19
90	0.61
120	3.07
133	3.66

Supplementary Note 1. Spin torque ferromagnetic resonance measurements

The applied radiofrequency (RF) current (I_{RF}) to the $TaIrTe_4$/Py device generates a spin current due to the spin orbit coupling of the spin source layer ($TaIrTe_4$) and this generated spin current then injected into the adjacent Py layer and induce a SOT. The SOT then result in the oscillation of the resistance of the device due to the anisotropic magnetoresistance of the Py layer. The mixing of the I_{RF} and oscillating resistance can be measured as a *dc* voltage signal (V_{mix}) using a lock-in-amplifier. The V_{mix} from STFMR can be expressed as given in equation (1) of the manuscript.

The charge-to-spin conversion efficiency ($\xi_{DL,k}$) due to the *y* and *z* polarized (σ_y and σ_z) spin current can be obtained using lineshape analysis[1-3]

$$\xi_{DL,y} = \frac{J_{\sigma y}}{J_c} = \frac{S_p - S_n}{A_p + A_n}\frac{e\mu_0 M_S t_{FM} t_{SH}}{\hbar}\left[1 + \left(4\pi M_{eff}/H\right)\right]^{1/2} \quad (1)$$

$$\xi_{DL,z} = \frac{J_{\sigma z}}{J_c} = \frac{A_p - A_n}{A_p + A_n}\frac{e\mu_0 M_S t_{FM} t_{SH}}{\hbar}\left[1 + \left(4\pi M_{eff}/H\right)\right]^{1/2} \quad (2)$$

where t_{FM} and t_{SH} are the thickness of the Py and $TaIrTe_4$ layer, respectively. S_p (A_p) and S_n (A_n) are the S (A) at positive and negative H, respectively.

Supplementary Note 2. Estimation of damping-like torque efficiencies using STFMR measurements with spin polarizations along *x*, *y*, and *z* directions

By fitting Eq. 3 and 4 to the extracted values and considering that A_{FL}^{Y} is due to the Oersted field alone, the amplitudes of the damping-like torque efficiencies per unit current density in the $TaIrTe_4$ layer can be defined as,[1,4]

$$\xi_{DL}^{X} = \frac{S_{DL}^{X}}{A_{FL}^{Y}}\frac{e\mu_0 M_S t_{TaIrTe4} t_{FM}}{\hbar}\sqrt{1 + \left(\frac{M_{eff}}{H_a}\right)} \quad (3)$$

$$\xi_{DL}^{Y} = \frac{S_{DL}^{Y}}{A_{FL}^{Y}}\frac{e\mu_0 M_S t_{TaIrTe4} t_{FM}}{\hbar}\sqrt{1 + \left(\frac{M_{eff}}{H_a}\right)} \quad (4)$$

$$\xi_{DL}^{Z} = \frac{A_{DL}^{Z}}{A_{FL}^{Y}}\frac{e\mu_0 M_S t_{TaIrTe4} t_{FM}}{\hbar} \quad (5)$$

here, $t_{TaIrTe4}$ is the thickness of the $TaIrTe_4$ layer.

Supplementary Note 3. Details of second-harmonic Hall analysis

The second harmonic Hall voltage can be expressed as,[3,5,6]

$$V_{2w} = D_{DL}^{Y} cos\phi + D_{DL}^{X} sin\phi + D_{DL}^{Z} cos2\phi + F_{FL}^{Y} cos\phi\, cos2\phi + F_{FL}^{X} sin\phi\, cos2\phi + F_{FL}^{Z} \quad (6)$$

with,

$$D_{DL}^{Z} = -\frac{\tau_{DL,0}^{Z}}{\gamma}\frac{V_P}{H_{ext.}} \quad (7)$$

Here, the damping-like torque produced by spins in the Z direction is, $\tau_{DL,Z} = \tau_{DL,0}^{Z}\hat{m} \times (\hat{Z} \times \hat{m})$. V_P is the coefficient of the peak planer Hall effect voltage, $V_{PHE} = V_P sin2\phi$, for the given amplitude of alternating current, and V_A is the coefficient of the peak anomalous Hall voltage, $V_{AHE} = V_A cos\theta$. The damping-like torque efficiencies per unit electric field associated with each component k are,

$$\theta_{DL}^{Z} = \frac{2e}{\hbar}\frac{\mu_0 M_S t_{FM}}{\gamma}\frac{\tau_{DL,0}^{k}}{E} \quad (8)$$

where, M_S is the saturation magnetization, t_{FM} is the thickness of the ferromagnetic layer (Py), e is the electronic charge, $\hbar$ is the Plank's constant, and E is the peak electric field associated with the alternating current.

Since $\mu_0 H_k \approx 0.75\ T$ for the samples in this work and anomalous Hall voltage V_A is relatively small, the in-plane torque contributions to $V_{2\omega}$is small which make these components difficult to measure accurately using this technique. For the harmonic Hall measurements, the focus is on the measurement of out-of-plane damping-like torque (θ_{DL}^{Z}) efficiency.

Supplementary References

1.Liu, L., Moriyama, T., Ralph, D. C. & Buhrman, R. A. Spin-torque ferromagnetic resonance induced by the spin Hall effect. Phys. Rev. Lett. 106, 036601 (2011).

2.Shi, S. et al. All-electric magnetization switching and Dzyaloshinskii–Moriya interaction in WTe2/ferromagnet heterostructures. Nat. Nanotechnol. 14, 945–949 (2019).

3.MacNeill, D. et al. Control of spin–orbit torques through crystal symmetry in WTe2/ferromagnet bilayers. Nat. Phys. 13, 300–305 (2016).

4.Bose A, Schreiber NJ, Jain R, Shao D-F, Nair HP, Sun J *et al.* Tilted spin current generated by the collinear antiferromagnet ruthenium dioxide. *Nature Electronics* 5, 267-274 (2022).

5.Hayashi M, Kim J, Yamanouchi M, Ohno H. Quantitative characterization of the spin-orbit torque using harmonic Hall voltage measurements. *Phys Rev B Condens Matter* 2014; **89**: 144425.

6.Avci CO, Garello K, Gabureac M, Ghosh A, Fuhrer A, Alvarado SF *et al.* Interplay of spin-orbit torque and thermoelectric effects in ferromagnet/normal-metal bilayers. *Phys Rev B Condens Matter* 2014; **90**: 224427.
